# Rare occurrence of severe blindness and deafness in Friedreich ataxia: a case report

**DOI:** 10.1186/s40673-021-00140-6

**Published:** 2021-07-15

**Authors:** Joana Damásio, Ana Sardoeira, Maria Araújo, Isabel Carvalho, Jorge Sequeiros, José Barros

**Affiliations:** 1 Department of Neurology, Hospital de Santo António, Centro Hospitalar Universitário do Porto, Largo do Professor Abel Salazar, 4099-001 Porto, Portugal; 2 UnIGENe and CGPP, IBMC – Institute for Molecular and Cell Biology, i3S – Instituto de Investigação e Inovação em Saúde, Universidade do Porto, 4200-135 Porto, Portugal; 3 Department of Ophtalmology, Hospital de Santo António, Centro Hospitalar Universitário do Porto, 4099-001 Porto, Portugal; 4 Department of Otorhinolaryngology, Hospital de Santo António, Centro Hospitalar Universitário do Porto, 4099-001 Porto, Portugal; 5 ICBAS - Instituto de Ciências Biomédicas Abel Salazar, Universidade do Porto, 4050-313 Porto, Portugal

**Keywords:** Friedreich ataxia, Blindness, deafness

## Abstract

**Background:**

Friedreich ataxia is the most frequent hereditary ataxia worldwide. Subclinical visual and auditory involvement has been recognized in these patients, with co-occurrence of severe blindness and deafness being rare.

**Case report:**

We describe a patient, homozygous for a 873 GAA expansion in the *FXN* gene, whose first symptoms appeared by the age of 8. At 22 years-old he developed sensorineural deafness, and at 26 visual impairment. Deafness had a progressive course over 11 years, until a stage of extreme severity which hindered communication. Visual acuity had a catastrophic deterioration, with blindness 3 years after visual impairment was first noticed. Audiograms documented progressive sensorineural deafness, most striking for low frequencies. Visual evoked potentials disclosed bilaterally increased P100 latency. He passed away at the age of 41 years old, at a stage of extreme disability, blind and deaf, in addition to the complete phenotype of a patient with Friedreich ataxia of more than 30 years duration.

**Discussion:**

Severe vision loss and extreme deafness has been described in very few patients with Friedreich ataxia. Long duration, severe disease and large expanded alleles may account for such an extreme phenotype; nonetheless, the role of factors as modifying genes warrants further investigation in this subset of patients.

**Supplementary Information:**

The online version contains supplementary material available at 10.1186/s40673-021-00140-6.

## Background

Friedreich ataxia (FRDA) is the most frequent hereditary ataxia worldwide. The underlying genetic mechanism is, in the majority of patients, an unstable, pathological GAA expansion in *FXN* gene leading to decreased expression of frataxin, a ubiquitous mitochondrial protein [1]. First symptoms usually appear around puberty, and cardinal neurological features include cerebellar ataxia, sensory loss, pyramidal signs and absent reflexes in lower limbs [2]. Atypical phenotypes, with retained reflexes, late or very late onset have been thoroughly characterized for the last years [3]. The most frequent non-cerebellar symptoms encompass cardiac repolarization abnormalities, cardiomyopathy, scoliosis and urinary system disorders [2, 4]. Optic and auditory involvement may also be present in FRDA, with most patients having only mild or no symptoms at all [2, 4, 5]. Here we report the rare occurrence of blindness and deafness as part of an extreme FRDA phenotype.

## Case presentation

### A) Methods

This patient was part of a prospective study on hereditary cerebellar ataxias approved by the Institutional Ethics Committee. The patient had been followed at our hospital since 1998; and, from 2016 to 2019, he was evaluated through a structured protocol, comprising definition of age-at-onset of the various neurological symptoms/ signs, the Scale for the Assessment and Rating of Ataxia (SARA) and the Inventory of Non-Ataxia signs (INAS). Every 6 months a questionnaire on new neurological symptoms and the INAS was applied, as well as a complete neurological exam, comprising SARA. The missing clinical information was obtained through a detailed review of medical records and video files. Brain MRI was performed in 1.5 T scanner. DNA was collected from peripheral blood of patient and parents, and stored at CGPP-IBMC, i3S.

### B) Case report

**Fig. 1:**
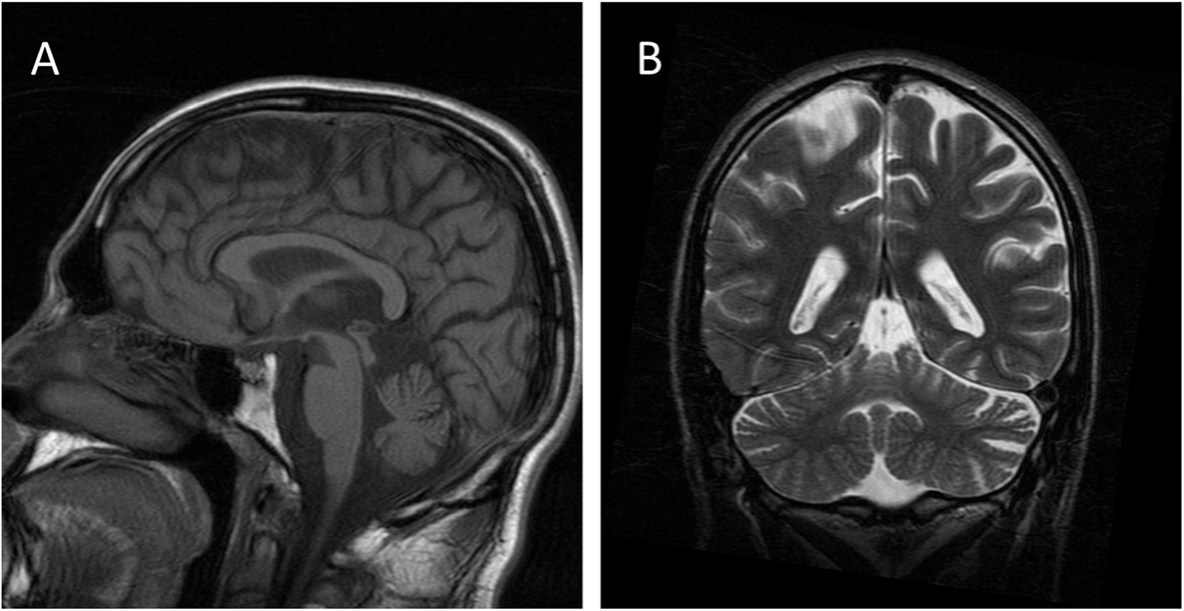
Brain MRI T1 (**A**) and T2 (**B**) sequences showing atrophy of vermis and cerebellar hemispheres

This was a male patient, homozygous for a 873 GAA expansion in the *FXN* gene, who presented gait instability by age 8 years. Dysarthria and upper limbs dysmetria emerged subsequently, and 4 years after disease onset he was wheelchair-bound. Scoliosis was diagnosed at age 16, and cardiomyopathy detected 1 year later. When 22 years-old he complained of bilateral hypoacusia, progressing to severe deafness over 11 years. By age 26, visual impairment was noticed and rapidly deteriorated: visual acuity decreased to 0.4 in just 1 year, and further decreased to light perception in the following 2 years. At that time, fundoscopic exam showed marked pallor of the optic discs, with sharp edges and normal retina. There was no history of exposure to drugs or toxins that could affect vision or hearing. Diabetes was diagnosed in his early thirties. By the age of 40 he presented scandid dysarthria, blindness, decreased amplitude of horizontal saccades, severe deafness impairing communication, tetraparesis predominantly affecting the lower limbs, absent deep tendon reflexes, absent position sense in the halluces, and truncal and finger-to-nose ataxia, scoring 38 on SARA (video). Even though a neuropsychological assessment could not be performed, there was no evidence of cognitive deterioration. He passed away with 41 years, after severe pneumonia. By the time visual and hearing impairment were identified, a thorough investigation was conducted: haemogram, chemistry, serology and immunology studies were normal. Genetic variants associated with Leber hereditary optic neuropathy (LHON) were excluded. Visual evoked potentials disclosed bilaterally increased latency (right eye: 144 milliseconds, left eye: 148 milliseconds) and decreased amplitudes of P100. Audiograms, performed over a period of 15 years, documented progressive sensorineural deafness, most striking for low frequencies (supplementary figure). On brain MRI (at age 29 years old), there was cerebellar, pons and medulla atrophy, with optic nerves exhibiting normal thickness and signal (Fig. 1).


**Additional file 1: Video Segment 1** The patient presents scandid dysarthria. Throughout the video, both the doctor and his mother have to speak very loudly for him to hear. Mild involuntary head movements, suggestive of titubation, as well as involuntary eyebrow movements, are present. **Segment 2** We observe mild head movements, when initiating horizontal saccades. Also, saccades are slow and with restricted horizontal range. On both video segments, there are involuntary eye movements of a roving nature, probably due to the absence of fixation, as is frequently observed in blind patients.

## Discussion

The first description of optic atrophy in FRDA was made by Sjögren, in 1940, in 12% of his patients [2]. Four decades later, Harding identified optic atrophy in 30% of cases: only 5.2% with severely and 13% with mildly reduced visual acuity, the remaining being asymptomatic [2]. Deafness was present in 7.8%, ranging from severe (0.9%) to mild (5.2%) [2].

Across the years, a few studies have demonstrated that subclinical involvement of visual and auditory systems is much more frequent than severe blindness and deafness [5–7]. Reduced visual acuity has been identified in 3.1–13% [4, 6, 8], and increased latency of visual evoked potentials in a much higher percentage of individuals: 34–70% [5, 6, 9]. In a study conducted by Fortuna et al., all the 26 individuals had thinning of the retinal nerve fibre layer on optical coherence tomography, in spite of only five having reduced visual acuity [5]. Involvement of posterior visual pathways was also present in these patients, with significantly higher apparent diffusion coefficients on diffusion-weighted MRI of the optic radiations [5]. Hearing loss of variable severity has been reported in 10–39% [4, 6, 8], with abnormal conduction in central pathways identified in 61–100% [6, 8, 10]. Impaired speech-understanding at levels of everyday background noise has been reported in up to 90% of patients, developing as soon as early-school years [7, 10].

Notwithstanding the relative frequency of subclinical visual and auditory impairment, co-occurrence of severe blindness and deafness are rather uncommon, but may be clustered with diabetes [2]. Critical vision loss resembling LHON has been described in very few patients, most being compound heterozygotes, with large expanded alleles, long duration and advanced disease [5, 11, 12]. The patient here reported had a catastrophic and rapidly progressive optic neuropathy, while hearing loss had a protracted course over 11 years until reaching a stage of critical deafness. Opposed to what has been described he was not a compound heterozygote, but a large expansion and long disease duration could partly account for such a severe phenotype.

Underlying pathophysiological mechanisms are incompletely understood. Current lines of evidence suggest anterior and posterior visual pathways degeneration; whereas in auditory function, the cochlea appears to be spared and central auditory brainstem is affected [5, 7]. A study on a mouse model, specifically addressing retinal ganglion cell death, suggested that optic atrophy was a consequence of increased sensitivity to oxidative stress, due to impaired intra-cellular iron regulation [13]. Frataxin deficiency may impact the respiratory chain, leading to mitochondrial iron deposition and dysfunction with consequent increased cellular susceptibility to oxidative stress [5, 13].

With this report we wish to (1) further contribute to the characterization of optic and auditory involvement in FRDA, (2) stress the need to better understand the underlying mechanisms, as well as their genetic and epigenetic modifying factors (including somatic heterogeneity); and (3) increase awareness for this rare extreme phenotype. Improved healthcare services and longer survival of early-onset patients will probably increase the frequency of these devastating manifestations.

## Supplementary Information

**Additional file 2: Supplementary figure**. audiograms documenting progressive sensorineural deafness (b: 2003, c: 2018).

## Abbreviations

CGPP: Centro de Genética Preditiva e Preventiva
DNA: Deoxyribonucleic acid
FRDA: Friedreich ataxia
FXN: Frataxin
IBMC: Instituto de Biologia Molecular e Celular
INAS: Inventory of Non-Ataxia signs
MRI: Magnetic Resonance Imaging
SARA: Scale for the Assessment and Rating of Ataxia
