# Supplementary figures and images for: Rare occurrence of severe blindness and deafness in Friedreich ataxia: a case report
Source: Cerebellum Ataxias. 2021 Jul 15;8:17. doi: 10.1186/s40673-021-00140-6 (PMC8283931; doi:10.1186/s40673-021-00140-6)

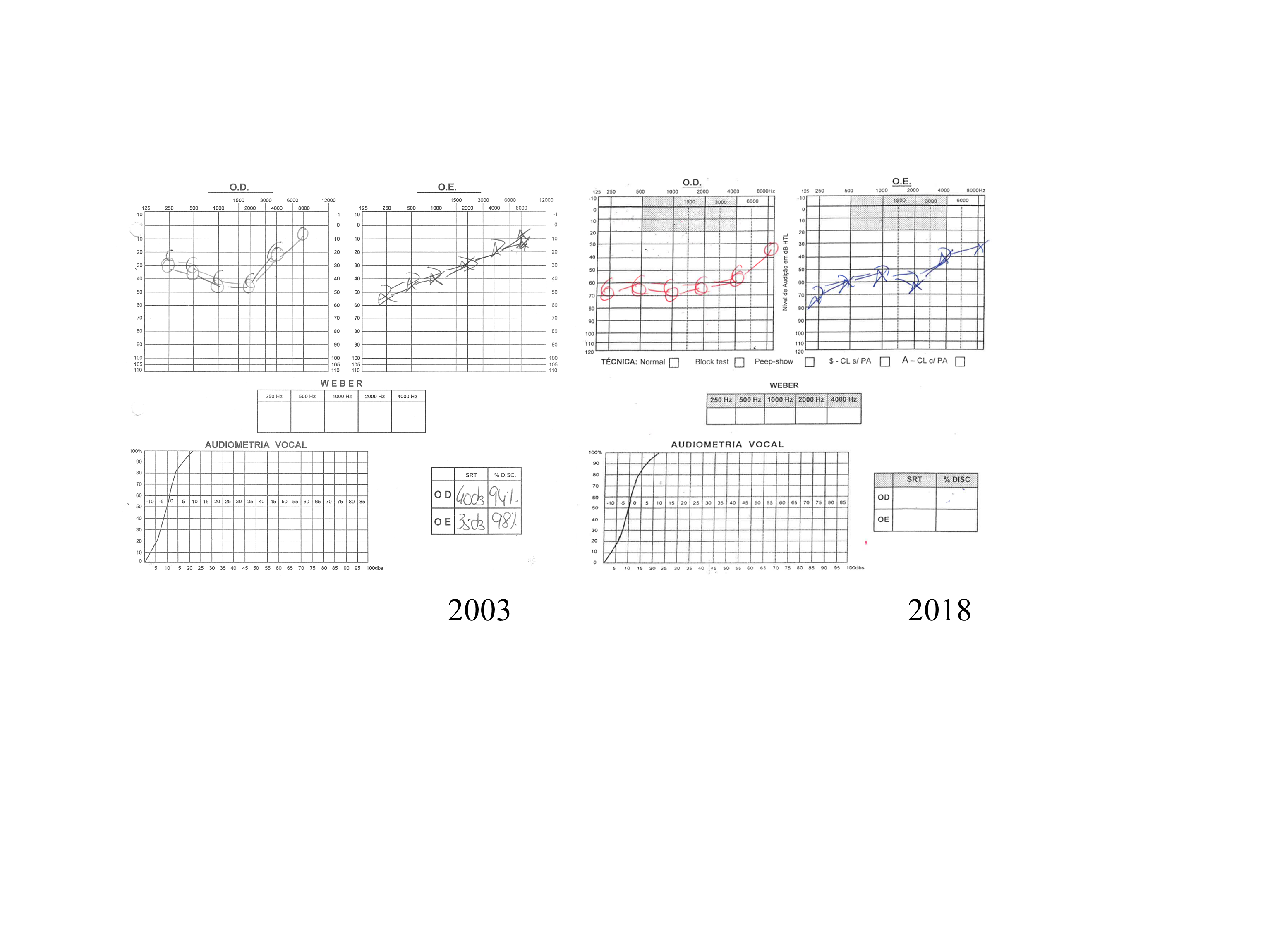

Supplement: Supplementary file 2 — Additional file 2: Supplementary figure. audiograms documenting progressive sensorineural deafness (b: 2003, c: 2018). [file 40673_2021_140_MOESM2_ESM.tif]
